# Surgical Treatment of Intramedullary Hemangioblastomas: Current State of Problem (Review)

**DOI:** 10.17691/stm2021.13.5.10

**Published:** 2021-10-29

**Authors:** S.Yu. Timonin, N.A. Konovalov

**Affiliations:** PhD Student N.N. Burdenko National Medical Research Center for Neurosurgery, Ministry of Health of the Russian Federation, 16, 4^th^ Tverskaya-Yamskaya St., Moscow, 125047, Russia;; Professor, Corresponding Member of the Russian Academy of Sciences, Head of 10^th^ Neurosurgery Department; Deputy Director for Science N.N. Burdenko National Medical Research Center for Neurosurgery, Ministry of Health of the Russian Federation, 16, 4^th^ Tverskaya-Yamskaya St., Moscow, 125047, Russia

**Keywords:** spinal cord hemangioblastoma, preoperative embolization, intraoperative imaging, indocyanine green, vascular tumor diagnosis

## Abstract

Intramedullary hemangioblastomas (HAB) refer to very rare highly vascularized vascular spinal cord tumors associated with various neurological disorders. Effective HAB therapy to a greater extent depends on diagnostic accuracy and the absence of intra- and postoperative complications.

The present study is a review of publications concerned with modern diagnostic and therapeutic techniques to control spinal HAB. The authors showed that perfusion computed tomography, computed tomographic angiography, and magnetic resonance angiography can be reasonably used for diagnosis and differentiation in a number of HAB due to their high vascularization. Preoperative embolization significantly reducing intraoperative bleeding risks is highly efficient. Some authors recommend this procedure in case of large lesions and high risks of intraoperative bleeding. The review also considered intraoperative imaging of a tumor and its feeding vessels using indocyanine green providing inspectability over the total tumor resection and clear imaging of tumor vascular architecture. The advantages and restrictions of the mentioned procedures were described.

## Introduction

Hemangioblastomas (HAB) are histologically benign vascular tumors referring to grade I tumors according to WHO classification [1]. HAB development can be sporadic (60%) or related to von Hippel–Lindau (VHL) syndrome (40%). Revealing multiple HAB in over 90% of cases is due to the presence of tumor suppressor gene mutations [2]. In 50–70% of cases, HAB are associated with cysts [3]. A mass, as a rule, is well delineated, round, small-sized, consisting of a dense network of thin-walled blood vessels lined with squamous endothelial cells. Between vessels, there are interstitial cells, the cytoplasm of which has an increased amount of lipids. Depending on a developing component (stromal or vascular) prevailing, there can be distinguished cellular and reticular histological HAB variants [4].

Hemangioblastomas develop mainly in infratentorial structures, such as the cerebellum, the brain stem, and the spinal cord. Primarily, they are located in the posterior cranial fossa (83%). Their frequency in the spinal cord reaches 13% [5].

Spinal hemangioblastomas refer to very rare tumors: over the period 2000–2015, less than 100 HAB cases were reported in literature [6]. In general, they account for 3% of all diagnosed CNS tumors and 2–6% of spinal cord lesions [1].

In most cases, HAB are located in the dorsal spine, it is accompanied by the growth of sensation disorders below the solid or cystic tumor component [7]. Among motor defects, there are hyperreflexia, strength diminution in limbs. At late stages, there can be intestinal and urinary malfunctions [8]. Sensory disorders and pain are present rather frequently and related to tumor-associated dermatomes, cysts, and syringomyelias [9]. A clinical presentation of a tumor depends on its size, localization, as well as the extent of exposure on the spine. Despite tumor high vascularization, the risk of subarachnoid hemorrhage is extremely low.

The main treatment method in HAB is microsurgical tumor resection under neurophysiological monitoring, and its success to a greater degree depends on diagnostic accuracy, preoperative imaging of tumor vascular anatomy, and a correct operative plan.

## Diagnosis

For CNS tumors including HAB, a variety of imaging modalities are used.

### Magnetic resonance imaging (MRI)

Currently, the gold standard of instrumental diagnosis of spinal tumors is MRI. The methods used in clinical practice prior to MRI could hardly provide a clear-cut idea of a tumor process and its growth in the spinal cord. Whereas MRI enables to image not only tumors but also such structural changes of the spine as syringomyelic cysts, hemorrhages, and brain edemas [10].

In addition to focal changes of a magnetic resonance signal from cerebrospinal substance, MRI diagnosis of intramedullary lesions is based on three basic imaging characteristics [10]. First, it is a local or diffuse increase in spinal cord volume [11]; in its absence, the following possible non-tumor causes of myelopathy such as demyelinating processes, vascular malformations, sarcoidosis, etc. should be taken into account [12]. Secondly, most intramedullary tumors (even benign) are prone to accumulate gadolinium-containing contrast agents, i.e. there should be contrast enhancement [13]. However, the absence of contrast in an increased volume of the spinal cord cannot exclude a neoplastic process [14]. One more diagnostic character is the presence of cysts [10].

As key diagnostic characteristics, HAB has the combination of cystic changes of the spinal cord with a well-delineated solid node, and the presence of dilated tortuous vessels in the subarachnoid space (since this tumor type is characterized by high vascularization) [15]. Moreover, in case of VHL syndrome, an important criterion is multiple solid tumor nodes.

**Figure 1.**
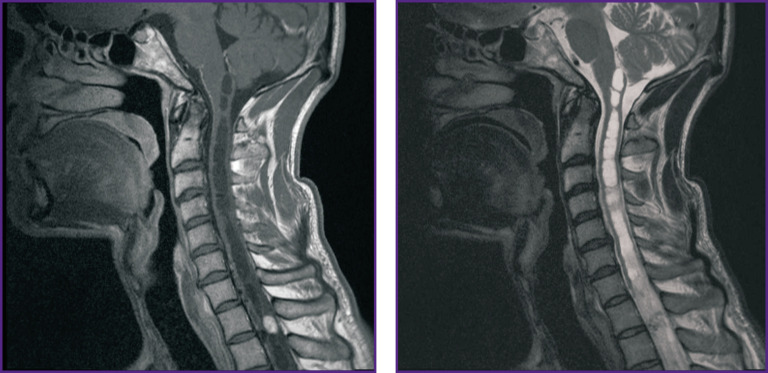
MRI of a patient with intramedullary hemangioblastoma at Th_1_–Th_2_ level (source: authors)

Tumor MRI, as a rule, is characterized by iso- or hypointense signal on Т1-WI [10]. On Т2-WI, the signal is sharply hyperintense with flow voids, which can appear due to the fact that there are vessels with fast blood flow in a tumor [16]. In most cases, spinal HAB are characterized by syringomyelic cysts located above or below a solid component [16], but in some cases, tumors are cyst-like and have a small mural solid component, which is more typical for their cerebral localization [17]. After contrast administration, there is significant enhancement of magnetic resonance signal in a tumor revealing its clear boundaries. Small HAB usually demonstrate homogeneous contrast accumulation, while large tumors can be inhomogeneous due to cysts, intratumor hemorrhage foci, as well as dilated and tortuous vessels [18] (Figure 1). Signal intensity from cysts can vary: high-protein cysts formed due to intra-node hemorrhages or tumor transudation have high-intensity signals [19]. If there are no cysts, there can be spinal edema above and below the tumor.

### Perfusion computed tomography (CT)

Perfusion CT can be used to reliably differentiate HAB from non-vascular masses, as well as estimate their blood supply intensity. Such study is based on mathematical analysis of the data obtained through a series of sequential scanning of single-level tissues by a contrast agent passing through them. When scanning, there being successively measured tissue density at every section point; the obtained values are proportional to the concentration change of a contrast agent passing through a contrast section. The use of special software enables to calculate quantitative parameters of blood microcirculation, estimate blood flow volume and its dynamic changes, as well as plot perfusion maps for visual analysis; such maps show characteristic blood supply of the tissues under analysis using color indication. The maps enable to promptly determine the areas with pathological changes.

**Figure 2.**
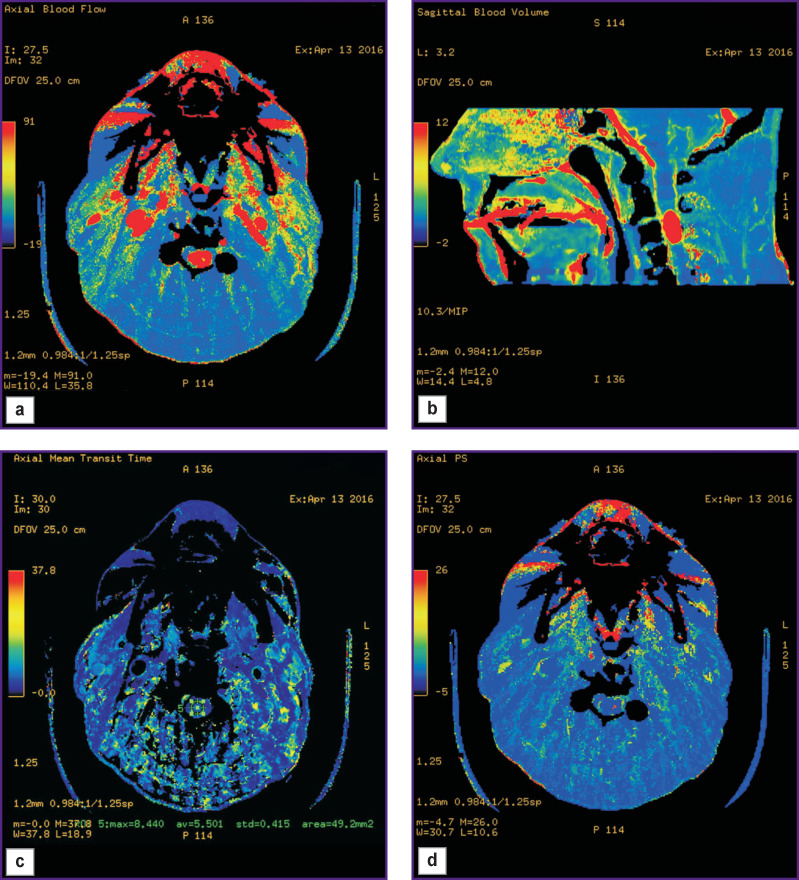
Perfusion maps of a patient with intramedullary hemangioblastoma at С_3_ level in CBF (а), CBV (b), MTT (c), PS (d) modes (source: authors)

Perfusion CT can be of use for HAB preoperative differential diagnostics, especially in case of solid structure tumors. CBV and CBF values, determinable in HAB, appear to be the highest among intra- and extracerebral infratentorial tumors [20]. Leonov et al. [21] demonstrated HAB of the posterior cranial fossa to be the area of increased perfusion compared to brain matter. Informativity analysis of using CT, MRI, direct vertebrobasilar angiography, MR-angiography, and perfusion CT showed the capability to obtain extra information on hemodynamic changes in a tumor using perfusion CT, as well as the efficiency of applying the technique for HAB differential diagnostics from other contrasted lesions [22]. According to clinical recommendations on diagnosis and treatment of intramedullary spinal tumors, perfusion CT (Figure 2) is a method of choice if differential diagnostics is necessary among the groups of intramedullary tumors of the spinal cord, and to exclude demyelinating diseases [23].

*Computed tomographic angiography (CTA)* is a technique, which enables to have 3D CT image of vascular anatomy in the tumor area. It promotes rational planning of a surgical intervention for vascular tumor removal, decrease of intra- and postoperative complications, as well as provides more favorable prognosis [24, 25]. Moreover, the average duration of CTA is short: e.g., [26] reported the average duration of 3D CTA compared to MRI when studying spinal tumors to be only a minute, and data post-processing was 30 min.

**Figure 3.**
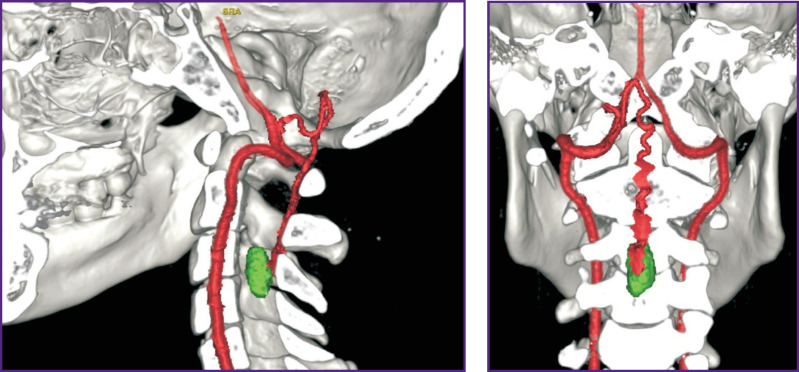
3D reconstruction based on CTA of intramedullary hemangioblastoma vascular anatomy at С_3_–С_4_ level (source: authors)

Computed tomographic angiography for HAB diagnosis was started using as early as in the 70–80s, ХХ century [27]. Researchers noticed its higher sensitivity and specificity compared to CT as related to the identification and characterization of HAB nodes. It frequently provides capability of precise diagnosis and detection of even small tumors. 3D CTA enables to visualize tumor anatomy and location with regard to the spine and feeding vessels. The technique is also informative for postoperative studies of spinal HAB [26] (Figure 3).

The drawbacks of the method are the following: possible allergic responses to a contrast agent. In addition, according to some authors, in case of spinal HAB, the technique fails to provide sufficient resolution if a tumor is located in the thoracic area; it is due to the surrounding structures [28, 29].

*Magnetic resonance angiography (MRA)* is one of the most advanced diagnostic techniques for structural and functional changes of the vascular system, it enables to clearly visualize the vascular anatomy of intact and tumor tissues, as well as determine blood flow functional characteristics. MRA can be with or without contrast. The main variants of the technique are time-of-flight and phase-contrast MRA. Time-of-flight MRA requires less time, so it is more frequently used, chiefly, for clinical diagnosis, especially in the pathology of intracranial arteries [30]. However, the technique fails to provide sufficient image quality of vessels with a slow blood flow. Phase-contrast MRA enables selectively visualize vessels with a fast or slow blood flow, as well as obtain the information on flow direction. It is rather effective to study blood supply of spinal vascular malformations; however, it is highly sensitive to turbulence that can result in artifacts [31].

**Figure 4.**
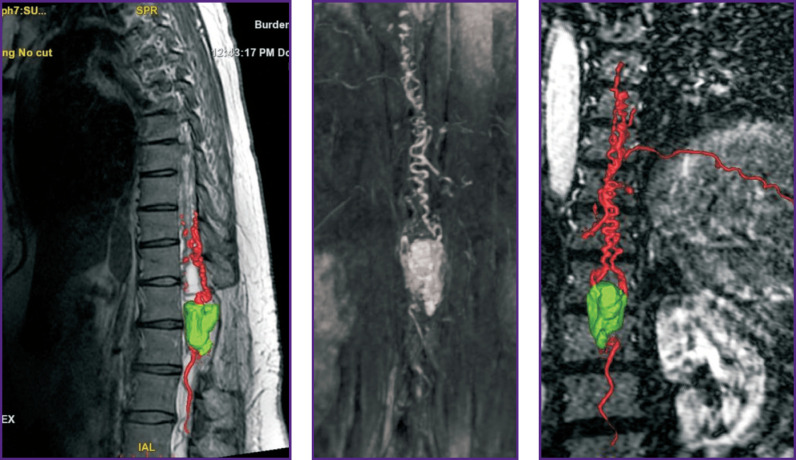
МRI-TRIKS with reconstruction (a patient with intramedullary hemangioblastoma at Th_12_ level) (source: authors)

Contrast MRA is mainly used to diagnose vascular tumors including HAB since without contrast enhancement this method does not give a comprehensive idea of HAB blood supply degree and source [4]. MRA enables to differentiate vascular tumors from other malformations, as well as visualize contrast movement. So, Binkert et al. [32] demonstrated differentiation validity using MRA of such spinal vascular diseases as arteriovenous malformations and fistulas, teratomas, and hemangiomas. Crisi et al. [33] showed that using MRA it is possible to trace a blood flow in a tumor starting from an early arterial and finishing at a late venous phase, and determine tumor vascular nature, its vascularization degree, as well as blood supply characteristics. Moreover, at a late venous stage, the technique enables to reveal the separation of solid and cystic tumor components (Figure 4).

## Surgical treatment

A generally accepted standard used to treat patients with intramedullary HAB is eradication of tumor under neurophysiological control; furthermore, the less marked are neurological disorders associated with a tumor by the beginning of an operation, the more favorable are the prognoses related to the recovery of patient’s functional condition in a postoperative period [34].

HAB management has a number of distinguishing features related to their blood supply characteristics. Due to high vascularization, HAB surgeries are carried out using microsurgical techniques aiming at complete tumor removal and preserving surrounding spinal tissues intact. Incomplete tumor resection can be carried out in case it is located in the anterior columns of the spinal cord that makes the approach difficult, as well as the presence of dense adhesions with the anterior spinal artery or between the tumor fragments and the spinal cord.

When choosing the most suitable surgical approach, one should take into account the tumor localization, the presence of cysts associated with a tumor, or syringomyelia, and the presence of surrounding tissue edema. Preoperative imaging of a tumor and its vascular system is of great importance when forming an appropriate scenario; it is particularly significant in case of tumor ventral localization. An increased risk of intraoperative bleeding can be reduced by preoperative embolization and intraoperative imaging.

The Table shows the largest series of surgeries for HAB resections. In most cases, it was a total tumor resection: in >90% patients (see, e.g., [2, 26, 35–42]). Some series have an increased number of subtotal resections — 15.0–37.5% [18, 43–45]. Some authors reported subtotal tumor resection to frequently result in recurrences or new tumors [44, 45], and according to the study by Takai et al. [44], recurrent tumors do not correlate with the presence of VHL syndrome.

**Table:** Studies devoted to spinal hemangioblastomas

Reference	Sampling years	Patients/surgeries	Patients with von Hippel–Lindau disease	Functional status changes after surgery (% of cases)			Postoperative follow-up (months)
				Improvement	Unchanged	Aggravation	
[2]	1988–2011	14/15	0	53.3	33.3	13.3	12–276
[18]	1994–2006	9/9	5	33	67	0	4–75
[26]	2007–2011	92/102	32	41.3	43.5	15.2	24–78
[35]	1984–2008	108/156	108	6	80	14	6–250
[36]	1997–2011	17/17	11	23.5	76.5	0	2–165
[37]	2000–2014	16/17	0	12.5	6.25	81.25	6–144
[38]	1996–2014	21/23	0	76	n/a	n/a	17 (on average)
[39]	2000–2013	14/18	7	78.6	n/a	n/a	6–96
[40]	2000–2013	24/26	10	41.2	47	11.8	12–144
[41]	2005–2015	11/11	0	72.7	27.3	0	6–48
[42]	2012–2017	18/37	2	100	0	0	3–18
[43]	1985–2002	34/40	25	32	50	18 (all are VHL)	36–204
[44]	1988–2008	24/24	8	37	53	10	5–221
[45]	2003–2012	16/30	4	18.7	56.3	25	4–290
[46]	2001–2014	15/19	8	80	6	14	6–132
[47]	1984–1997	19/22	0	40.9	50	9.1	6–142
[48]	1988–1997	44/55	44	7	84	9	6–12
[49]	1986–2000	14/14	6	57.2	21.4	21.4	15–161
[50]	1990–2005	23/23	8	21.7	74	4.3	6–120
[51]	1991–2005	20/24	2	29.2	58.3	12.5	6–78
[52]	2002–2007	15/17	4	6.5	87	6.5	15–72
[53]	1995–2008	20/20	11	25	65	10	1–62
[54]	2000–2017	20/20	4	90		10	30.9 (on average)
[55]	2010–2018	18/18	16	94.5		5.5	n/a

Here: n/a — non-available.

During a short-term postoperative period, some authors observed a short decline of the neurologic status in some patients. So, long-term sensory dysfunctions were found in 57% of operated subjects [38]. The researchers link the findings to a comparatively brief postoperative follow-up (17 months, on average). According to [46], postoperative neurological disorders are revealed in 50–80% of patients, and in the long range, they continue to persist in 10%.

As the Table shows, an operative intervention resulted in patient’s neurological status improvement in the long range, in 40.8% of cases (6–100%), on average. The status was unaffected in 57.2% of patients (0–80%). The aggravation was revealed in 10% of cases (0–25%). Some authors relate patients’ postoperative aggravation to VHL syndrome [35, 43], incomplete tumor resection [26], or tumor ventral localization, or referring to a complete intramedullary type [35].

### Preoperative embolization is a technique to minimize hemorrhage risks

Uncontrolled intraoperative hemorrhage in HAB resection, which occurs due to the intense blood supply, is the main cause of unfavorable surgery outcomes [56]. The bleeding from tumor stroma, its feeding arteries, or draining veins is a risk factor in regard to possible hemorrhages to surrounding tissues or tumor residual (in incomplete tumor resections), edema, and impaired circulation. Significant surgery improving safety is provided by preoperative embolization, which enables to reduce the blood flow in a solid tumor component, therefore, decreasing the risk of intraoperative bleeding; in some cases, the procedure even enables to remove tumors, which were considered unresectable before [56]. Moreover, in case of spinal tumors, embolization can relieve the symptoms, reduce the spinal cord compression and, probably, slow the tumor growth without surgery [57].

Despite the above-mentioned positive effects, preoperative embolization can lead to a variety of undesirable complications due to the proximity of feeding arteries to the radicular medullar artery. Relative contraindications can include uncorrectable coagulopathy, renal failure [56]. An interval between embolization and a subsequent tumor resection should not exceed 3 days [58].

As embolic agents for spinal tumor therapy, the following ones are used: microparticles (polyvinyl alcohol (PVA), gelatin microspheres) or liquid embolic compounds (NBCA, Onyx). The most common embolic agent consisting of microparticles is PVA; however, its particles vary in size and surface shape and swell under a contrast agent that can result in their aggregation and catheter obstruction [59].

Liquid embolic agents provide prompt and permanent embolization, as well as deep penetration into a tumor. However, their usage requires certain experience and technical skills, and the results are difficult to control, it increasing non-target embolization risk that can lead to neurologic complications due to ischemic complications [60]. NBCA is a non-adsorbing agent able to polymerize promptly when in contact with blood or saline solutions. At the same time, these properties increase the risk of microcatheter obstruction, or feeding artery rupture when withdrawing a catheter [58]. Therefore, NBCA should be injected quickly and continuously, it making the delivery accuracy worse [61]. Onyx is administered more slowly, and its administration takes more time; it provides its complete penetration to tumor vessels and more precise control over embolization [62].

The analysis of publications over the period 1990–2015 [58] (37 articles describing >1300 cases of metastatic lesions, intradural and extradural tumors) showed that in 45.5% of surgeries described after 2008 (the year when Onyx was introduced), the procedure was performed using this agent.

Due to the rare nature of HAB in general, and spinal HAB in particular, as well as complicated anatomy of the arteries feeding these tumors, the experience of their preoperative embolization is still brief so far. A retrospective review [63] systemized the data on 29 cases of preoperative embolization of spinal HAB. Other publications reported on smaller series of patients under study (2‒7), the postoperative period varying from 1 to 161 months [35, 43, 45, 49, 51, 53, 64–66]. There should also be noted there is a report on 24 cases of preoperative embolization in spinal HAB in China [67].

In the above-mentioned studies, most patients after embolization underwent total tumor resection (e.g., 93% cases in the review [63], 86% — in [65], 100% — in studies [45, 49, 66]). The exceptions were two cases. The first one was subtotal resection due to intraoperative bleeding, its volume reaching 2.2 L [51]. In another case, the surgery turned out to be impossible due to embolic agent penetration to a tumor resulting in tumor consolidation; the patient was under the doctor’s observation [65].

Some authors report on a changing neurologic status according to McCormick classification after surgeries with preliminary tumor embolization. So, the study [49] reports 1 patient from the sampling of 4 patients not to change his status (IV→IV), while other cases demonstrated improvement (III→II, III→I, I→0). The article [51] showed a neurologic status to improve in 1 patient (0→I), while in other two it remained unchanged (0→0, III→III). In the study [53], the neurologic status did not change in 3 patients (I→I, I→I, II→II); in 2 patients, it improved (II→I, II→I). Finally, in the research [65], from seven patients with preoperative status I or II, six patients had no changes, while in one patient, the status improved (II→I). Other authors either give no information on neurologic status changes or confine themselves to mentioning the improvement only. No information was found on status aggravation after surgeries with embolization.

The authors of a number of articles report on the absence of complications related to embolization [35, 43, 45, 49, 64, 66, 68], while others remark about their rarity and temporal nature. So, from 29 cases considered in the review [63], only 3 patients had temporal complications including dysphagia (1), impaired sensitivity (1), and spasticity (1). In the long-term period (6–108 months), these symptoms disappeared completely. The analysis of 18 cases carried out in the study [65] showed that clinical complications found in 2 patients were temporary and associated with local edema and slight allergic reactions; the administration of corticosteroids enabled to normalize the situation. In addition, the authors described the series of 7 operations for spinal HAB resection with preliminary embolization. Two cases had complications; it caused surgery rescheduling. One patient developed slight allergic reaction, and the operation was performed 3 months later. The complication (vertebrobasilar infarction with subsequent unilateral cerebellar syndrome and gait disorder) in the second patient resulted in 4-month-surgery rescheduling. 2 patients were recorded to have slight embolic agent effusions beyond the target afferents, although the patients experienced no sequelae after the procedure. However, the subsequent tumor resection in one patient appeared to be impossible due to the fact that the tumor had hard consistency, and the spinal cord seemed to be edematous. The surgeon decided to discontinue the surgery to avoid severe neurologic impairments, and the patient was recommended to undergo regular clinical and radiological examinations. After 56-month follow-up, the tumor remained stable. Lee et al. [49] performed 4 embolization procedures. In one case only, the procedure failed due to a sudden partial occlusion of the left posterior inferior cerebellar artery that caused diplopia and speech disturbance. After procedure interruption, the symptoms disappeared. It should be noted that no lethal cases were reported in the above-mentioned studies. Most authors reported that the patients who had undergone embolization had less blood loss during the resection surgeries [63, 67]. One case was recorded to have massive intraoperative blood loss (2.2 L), and the doctors failed to prevent it by embolization [51].

Since the data on preoperative embolization efficiency and associated risks are frequently based on retrospective analyses of the surgeries performed using different materials, currently, there is no consensus on the procedure feasibility in the therapy of intramedullary HAB. Some authors hold the view of the procedure utility (if there are no direct contraindications) due to intraoperative blood loss decrease and overall surgery time reduction, as well as the increased chances for total tumor resection [35, 43, 45, 49, 66, 67, 69–73]; the others report about the possibility of developing complications associated with the procedure, such as intradural hemorrhage, spinal cord ischemia, as well as slight effuse of an embolic agent into a tumor, its thickening and complicating tumor resection [42, 53, 65, 74]. In general, available data suggest that if there are no direct contraindications, the procedure can be indicated for patients with a high risk of intraoperative bleedings, and in case of large tumors [63]. At the same time, according to idem authors, embolization gives no essential advantage in patients’ neurologic status change compared to microsurgical tumor removal. Thus, the procedure can be considered as a method enabling to ease a surgery and reduce associated risks rather than a method that can significantly improve a patient’s postoperative status.

### Intraoperative video monitoring

One more serious risk associated with spinal HAB resection, in addition to intraoperative bleeding, is a tumor solid component injury before imaging and exclusion of tumor feeding arteries. It can result in massive blood loss, sometimes reaching 3 L [75], as well as a permanent postoperative neurologic impairment frequently developing due to long-lasting attempts to stop bleeding using bipolar coagulation [76]. As a result, clear identification of feeding arteries and draining veins, as well as arterial supply exclusion before resection are essential conditions for safe and total (*en bloc*) resection.

**Figure 5.**
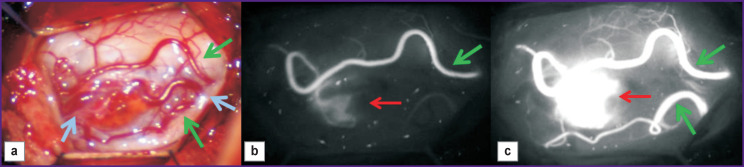
Intramedullary hemangioblastoma (source: authors): (а) intraoperative appearance (green arrows — feeding arteries; blue arrows — draining veins); video angiography: (b) 1^st^ second after tumor contrast enhancement (green arrow — the first feeding artery; red arrow — tumor solid component); (c) 3^rd^ second after tumor contrast enhancement (green arrows — feeding arteries; red arrow — actively contrasting solid tumor part)

One of possible solutions to the problem is intraoperative video monitoring using indocyanine green video angiography (ICG-VA) (Figure 5).

For the first time, ICG-VA was started using in the mid-70s in the ХХ century to perform ophthalmologic angiography. After that, the technology was being actively used for other researches including aneurysm and brain tumor surgery (see, for example, [77, 78]). Since 2011, the number of publications related to ICG-VA usage has started growing exponentially [79], however, currently, there are few works about effective application of ICG-VA in the surgery of intramedullary spinal tumors. The technology was found [80] to enable to control total intramedullary HAB resection. It is highly effective for imaging tumor feeding vessels in resection of a solid tumor component at С_7_–Th_2_ level [81]. Moreover, using ICG-VA it is possible to visualize dorsal HAB vessels at Th_12_ level, as well as control its total resection [82].

Hao et al. [83] reported about using ICG-VA in 7 patients with spinal HAB. Using the findings of the comparative analysis of pre-, intra-, and postoperative images the authors managed to locate feeding arteries and draining veins, as well as the tumor boundaries in 5 patients. Among other patients, in one case, a residual devascularized tumor was located deeply in the spinal cord parenchyma and appeared inaccessible for imaging; in the second case, only draining veins could be visualized, since the ventrolateral tumor was located below the spinal cord parenchyma. The surgeries performed enabled to completely remove six from seven tumors, and the seventh was resected partially, and none of the patients were found to have essential postoperative neurologic impairments. Thus, in case of ventral and deeply located tumors, ICG-VA capabilities turn out to be limited.

Takami et al. [84] described 14 cases of surgical treatment of patients with intramedullary tumors in the cervical and thoracic spine. None case had side effects or complications related to ICG-VA. The image quality was significantly better than in subtraction angiography. The authors consider the main advantage of the technique to be the capability to locate spinal arteries and veins and assess postoperative blood circulation in spinal veins, as well as clear differentiation of feeding arteries, a tumor, and draining veins. Their following study based on generalized experience of ICG-VA application in 48 patients with intramedullary spinal tumors concluded that ICG-VA is a safe and useful method enabling to achieve accuracy in tumor resection [85]. ICG-VA usage is more promising in surgery of highly vascularized tumors. So, the study by Molina et al. [76] found the combination of ICG-VA and preoperative digital subtraction angiography during the operation for HAD resection *en bloc* to contribute to accurate determination of tumor vascular architecture that enables to reduce blood loss up to 100 ml and ensure no changes in neuromonitoring system signals.

Thus, intraoperative video angiography using indocyanine green is a useful tool in HAB surgery providing real-time imaging of both: a tumor itself, and its feeding and draining vessels; moreover, it enables to avoid excessive blood loss and incomplete tumor resection. However, when making decisions on the method applicability, one should take into account the imaging complexity of feeding arteries situated inside a solid tumor component [83]. Most HAB are located on dorsal surface of the spinal cord [48], therefore, the imaging of their feeding arteries using ICG-VA is not a complicated problem. In case of tumors located on ventral spinal surface, the feeding is due to the arteries from the anterior spinal artery pool. In such situation, the use of ICG-VA can hardly influence the imaging degree of the feeding vessels, as well as the tumor itself. Moreover, if tumor fragments, which were not removed completely during the surgery turn out to be devascularized, the method fails to offer the opportunity to detect them; in this case, it is required to use MRI or intraoperative ultrasonography [83].

## Conclusion

Due to high vascularization of hemangioblastomas, in some cases for diagnostics and differentiation of spinal tumors, it is reasonable, in addition to MRI, to use such techniques as perfusion computed tomography (in case of tumor solid structure, for differential diagnosis of hemangioblastomas from other intramedullary spinal cord tumors in order to exclude demyelinating disease), computed tomographic angiography (to identify and characterize hemangioblastoma nodes, for imaging a tumor and feeding vessels) and magnetic resonance angiography (to differentiate hemangioblastomas from other vascular malformations, and for imaging a blood flow through a tumor).

Surgical resection of spinal cord hemangioblastomas can be accompanied by preoperative embolization to reduce intraoperative bleeding risks since embolization ensures relatively low intraoperative risks. Irrespective of differing points of view existing between different researchers concerning complication risks, the findings they are reporting suggest that the procedure can be indicated in case of large-size tumors, and high risks of intraoperative bleeding. Intraoperative imaging is a promising method to visualize a tumor and its feeding vessels using indocyanine green providing inspectability during total tumor resection and clear imaging of its vascular architecture; it contributes to operative blood loss minimization.
